# Effects of Dizocilpine, Midazolam and Their Co-Application on the Trimethyltin (TMT)-Induced Rat Model of Cognitive Deficit

**DOI:** 10.3390/brainsci11030400

**Published:** 2021-03-22

**Authors:** Marketa Chvojkova, Hana Kubova, Karel Vales

**Affiliations:** 1 Institute of Physiology of the Czech Academy of Sciences, Videnska 1083, 142 20 Prague 4, Czech Republic; Hana.Kubova@fgu.cas.cz (H.K.); Karel.Vales@nudz.cz (K.V.); 2 National Institute of Mental Health, Topolova 748, 250 67 Klecany, Czech Republic; 3 2nd Faculty of Medicine, Charles University, V Uvalu 84, 150 06 Prague 5, Czech Republic

**Keywords:** cognitive function, trimethyltin, hippocampus, NMDA receptor, GABA A receptor, dementia, combination therapy, Alzheimer’s disease, neurodegeneration, neuroprotection

## Abstract

Research of treatment options addressing the cognitive deficit associated with neurodegenerative disorders is of particular importance. Application of trimethyltin (TMT) to rats represents a promising model replicating multiple relevant features of such disorders. N-methyl-D-aspartate (NMDA) receptor antagonists and gamma-aminobutyric acid type A (GABA_A_) receptor potentiators have been reported to alleviate the TMT-induced cognitive deficit. These compounds may provide synergistic interactions in other models. The aim of this study was to investigate, whether co-application of NMDA receptor antagonist dizocilpine (MK-801) and GABA_A_ receptor potentiator midazolam would be associated with an improved effect on the TMT-induced model of cognitive deficit. Wistar rats injected with TMT were repeatedly (12 days) treated with MK-801, midazolam, or both. Subsequently, cognitive performance was assessed. Finally, after a 17-day drug-free period, hippocampal neurodegeneration (neuronal density in CA2/3 subfield in the dorsal hippocampus, dentate gyrus morphometry) were analyzed. All three protective treatments induced similar degree of therapeutic effect in Morris water maze. The results of histological analyses were suggestive of minor protective effect of the combined treatment (MK-801 and midazolam), while these compounds alone were largely ineffective at this time point. Therefore, in terms of mitigation of cognitive deficit, the combined treatment was not associated with improved effect.

## 1. Introduction

Dementias, exemplified mainly by Alzheimer’s disease, represent a serious worldwide problem. The rising numbers of patients, significant socioeconomic burden, and limited treatment options necessitate research into treatments targeting the disabling cognitive deficit. Amyloid beta is assumed to play a pivotal role in the pathogenesis of the disease. Besides other detrimental effects, amyloid beta seems to contribute to disturbance of the balance between excitation and inhibition in limbic structures, including hippocampus. These alterations of neurotransmitter systems may contribute to the cognitive deficit. Therefore, pharmacological approaches aiming at restoration of the neurotransmission balance by modulation of glutamatergic and GABAergic systems seem reasonable (reviewed in [1]).

A representative of antagonists of the glutamate receptor of the N-methyl-D-aspartate (NMDA) type, memantine, is already clinically used and is capable of reducing the worsening of clinical symptoms in patients with Alzheimer’s disease [2]. It was also proven to decrease the levels of Alzheimer’s disease associated proteins amyloid beta and tau in a triple transgenic mouse model, suggesting its disease-modifying potential [3]. Multiple publications corroborate promising effects of NMDA receptor antagonists on cognitive functions in genetic and non-genetic rodent models of Alzheimer’s disease [4,5,6], and neuroprotective effects in other animal models [7,8,9]. Generally, the neuroprotective effect lies in mitigation of glutamate excitotoxicity [9]. However, as NMDA receptors are necessary for physiological neurotransmission, many NMDA receptor antagonists can induce undesirable side effects [9,10].

The role of the modulation of the GABAergic system by various compounds including benzodiazepines in the treatment of dementias did not receive bigger attention until the last decade [1,11]. Benzodiazepines acts as positive allosteric modulators of GABA_A_ receptors, therefore facilitating the inhibitory effect of GABA [12]. Such action may possibly help to restore the balance between excitation and inhibition. Besides, GABA_A_ receptor potentiators, including benzodiazepines, possess beneficial neuroprotective properties in animal and in vitro models [13,14,15,16,17]. On the other hand, side effects may limit the use of these compounds [18].

Among animal models of neurodegenerative diseases associated with cognitive deficit, one possible approach is a systemic administration of trimethyltin (TMT), an organometallic compound inducing neurodegeneration in the limbic system, particularly in the hippocampus [19,20,21], and behavioral alterations, including cognitive deficit in various tasks [22,23,24,25,26] in laboratory rats. The effect of TMT is age-dependent, affecting older animals more profoundly [27]. Moreover, the model shares other typical features of neurodegenerative disorders, such as oxidative stress [25], microglia activation [28], mitochondrial dysfunction [29], progressive pattern of action [22], altered neurotransmission [23,30,31,32], and altered expression of multiple gene groups, including those relevant to Alzheimer’s disease, such as presenilin 1, presenilin 2, amyloid precursor protein, and tau [33,34]. These properties make the TMT model a promising tool for testing of treatment options targeting the disabling cognitive deficit associated with neurodegenerative disorders, particularly Alzheimer’s disease.

The precise mechanisms of the selective neurotoxicity of TMT are complex and not yet fully elucidated. Oxidative stress, calcium overload, and mitochondrial damage are most probably involved, although other phenomena like glutamate excitotoxicity are also considered, as reviewed by Geloso et al. [35]. Among other pathologies, alterations of neurotransmitter systems [32] involving increased extracellular levels of glutamate [30,31] and decreased levels of GABA in hippocampus [23] seem to be present. Correspondingly, therapeutic effects of drugs with various mechanisms of action (anti-inflammatory agents, antioxidants, or agents correcting altered neurotransmission) were described (for review see [36]).

NMDA receptor antagonists [37] or positive modulators of GABA_A_ receptors [38] are able to accomplish the desired alleviation of the TMT-induced cognitive deficit. The precise underlying mechanism is difficult to specify due to the complex nature of TMT action. It is supposed to lie mainly in correction of altered neurotransmission [36], since the neuroprotective potential of these compounds in the TMT model appears rather limited [39,40,41].

Due to the complex nature of Alzheimer’s disease, combined treatment is expected to represent a more suitable approach than monotherapy [1,42]. Such an approach may lead to increased therapeutic effects and an improved side effect profile [42], which would be of great benefit. Furthermore, given the current interest in so-called multi-target directed ligands, these findings can represent a potential basis for future drug development. It seems reasonable to suppose that co-application of NMDA receptor antagonists and GABA_A_ receptor potentiators might, by complementary mechanisms, help to restore the excitatory/inhibitory balance and lead to an increased effect. The evidence from models of different central nervous system disorders suggests that co-application of NMDA receptor antagonists with GABA_A_ potentiators may be associated with beneficial synergistic interactions [15,43,44,45].

To our knowledge, it remains to be elucidated whether similar beneficial interactions may also occur in the TMT model. Therefore, the purpose of this study was to investigate whether co-application of an NMDA receptor antagonist and GABA_A_ receptor potentiator would increase the intended anti-amnestic therapeutic effect in the TMT model. As a selective NMDA receptor antagonist, we used MK-801 [46], as a representative of GABA_A_ receptor potentiators of the benzodiazepine class, we chose midazolam; its advantage over other benzodiazepines lies in its water-solubility [47]. MK-801, midazolam, or their combination were repeatedly administered to the TMT-injected rats. Subsequently, we focused on the cognitive performance of the animals in Morris water maze (a hippocampus-dependent task [48]) and contextual fear conditioning (task involving hippocampus- and amygdala-associated processes [49,50]). We hypothesized that the co-application would be associated with increased cognitive effect. In addition, the neurodegeneration in dorsal hippocampus was histologically assessed.

## 2. Materials and Methods

### 2.1. Animals

Thirty-two adult male Wistar rats (10–11 weeks old, 380–490 g) purchased from the Institute of Physiology of the Czech Academy of Sciences (accredited breeding colony) were used. After one week acclimatization period, the experiment has been initiated. As TMT may induce aggression [22,25], the animals were single housed since the day of TMT administration in transparent plastic cages (20 × 25 × 40 cm). The cages were located in an air-conditioned animal room with constant temperature (22 °C), humidity (50–60%), and 12 h light/dark cycle (lights on: 06:00–18:00 h). Water and food were available ad libitum. All experiments were conducted in accordance with the guidelines of the European Union directive 2010/63/EU and Act No 246/1992 Coll., on the protection of animals against cruelty, and were approved by the Animal Care and Use Committee of the Institute of Physiology of the Czech Academy of Sciences and by the Central Committee of the Czech Academy of Sciences (approval number 136/2013, approved 3 October 2013). All efforts were made to reduce the number of animals and minimize suffering.

### 2.2. Drugs and Experimental Design

The rats were pseudorandomly allocated to five groups: Saline, TMT, TMT + MK-801, TMT + midazolam, and TMT + MK-801 + midazolam. On Day 0, animals were intraperitoneally injected with a single dose of TMT (trimethyltin chloride, #146498, Sigma-Aldrich, St. Louis, MO, USA; 8 mg/kg body weight, TMT weight expressed as total salt, 8 mg/mL, dissolved in 0.9% saline). Control animals (group saline) received a corresponding volume of saline. MK-801 at a dose of 0.1 mg/kg ((+)-MK-801 hydrogen maleate, #M107, Sigma-Aldrich, 0.1 mg/mL, dissolved in 0.9% saline), midazolam at a dose of 5 mg/kg (midazolam hydrochloride, 5 mg/mL, Dormicum, Roche, Prague, Czech Republic), or both MK-801 and midazolam were applied to animals from corresponding groups 30 min before the application of TMT on Day 0 and then on a daily basis until Day 11, while animals from groups saline and TMT received 0.9% saline. All drugs were administered intraperitoneally.

**Figure 1:**
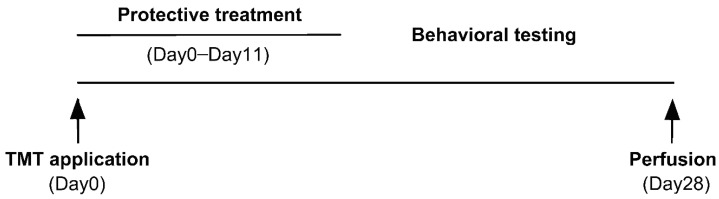
Timeline of the experiment.

The timeline of the experiment is shown in Figure 1. After finishing the treatment, a battery of behavioral tests was conducted. Ethical aspects were considered in the experimental design. The same animals were used for behavioral tests and histological assessment, enabling reduction of the number of animals. Perfusion was performed on Day 28 (therefore, after a 17-day drug-free period).

The number of animals in groups was: 7 animals in saline, 8 animals in TMT, 6 animals in TMT + MK-801, 6 animals in TMT + midazolam, and 5 animals in TMT + MK-801 + midazolam group.

### 2.3. Morris Water Maze

Morris water maze (MWM) was performed to assess cognitive performance. The apparatus consisted of a blue plastic circular pool (180 cm in diameter) with a circular platform (10 cm in diameter, transparent plastic, submerged 1 cm below the water surface). The position of the platform was constant (in the center of SW quadrant). The pool was filled with water (21–22 °C, 28 cm deep) colored by a small amount of non-toxic grey dye. The position of the rat was recorded every 40 ms by an overhead camera connected to a digital tracking system (Tracker, Biosignal Group, New York, NY, USA). The data was later analyzed using software Carousel Maze Manager 0.4.0 [51].

The MWM testing was initiated on Day 12 of the experiment and it was conducted during four consecutive days (MWM Day 1–4) in the light phase of the day. The rats were trained to find the hidden platform. Each day the rats underwent 8 swims from different starting points located on the periphery of the pool (in pseudorandom order). Animals were released into the water facing the inner wall of the pool. If the rat did not find the platform in 60 s, it was guided to the platform by the experimenter. On the MWM Day 4, after finishing the MWM sessions, the platform was removed, and the rats underwent a 30-s probe trial. Subsequently, the platform was returned to the pool, raised 1 cm above the water surface, and provided with a contrast rim for better visibility. A 60-s visible platform trial was conducted to assess sensorimotor functions and motivation [52].

The dependent variables for training trials were total distance (m) moved by the rat to reach the hidden platform (or total distance moved in the case of unsuccessful trials) and mean distance (cm) from platform (mean of the distances of the animal from the platform, sampled in 40 ms intervals), which represents a sensitive parameter, reflecting not only the ability to locate the platform, but also the search strategy [53]. Mean daily values were calculated for each animal and used for analysis. Latency was also analyzed, but as latency and distance are generally correlated, we report only the distance, which is less sensitive to possible differences in swimming speed. Cumulative latency (sum of all latencies during MWM Day 3 and 4) was calculated to assess the best achieved performance [54]. Days 3–4 were chosen because all groups reached asymptotic performance by MWM Day 3 (within each group, there was no significant difference between MWM Day 3 and Day 4 with respect to the distance moved as well as escape latency [latency data not shown]; two-way repeated measures ANOVA with Bonferroni post hoc test).

The dependent variables for probe trial and visible platform trial were dwell time in target quadrant (=the quadrant where the platform was originally located) and latency to find the platform, respectively.

“The periphery of the pool” refers to an 18-cm wide annulus; the wall of the pool represented the outer border of the annulus.

### 2.4. Contextual Fear Conditioning

Contextual fear conditioning is a cognitive task based on association of aversive stimulus with the context of its administration. In healthy animals, repeated exposition to the context leads to manifestation of freezing behavior [55]. The experiment was performed during two consecutive days (Day 17–18). An automated apparatus TSE Multi Conditioning System (TSE Systems, Bad Homburg, Germany) with corresponding software (TSE ActiMot) was used. The apparatus consisted of a testing box (44 × 44 cm; the floor was made of a stainless steel grid) and enabled administration of electric stimulus via the grid floor and detection of the animal’s activity using infrared sensors. For the first session (learning), the animal was placed into the apparatus and after 3 min, an electric stimulus (1 mA, direct current, 2000 ms) was administered. After 2 min, the stimulus was administered once more. Testing session was performed 24 h later. The animal was placed again into the testing box for 5 min and the cumulative duration of freezing was analyzed. Freezing episode was defined as absence of (other than breathing) body movements for 2 s or more.

### 2.5. Open Field Tests

The rats were subjected to open field tests to monitor their locomotor activity to exclude any presence of severe malaise or decreased state of well-being, possibly induced by the TMT toxicity and manifested as decreased/absent locomotion. These non-specific effects, if present, may influence the performance of the animals in the cognitive tests. Open field tests (10 min) were performed on Day 0 before TMT administration (baseline activity) and then weekly: on Day 7, Day 14, and Day 21. The TSE Multi Conditioning System (TSE Systems, Germany) apparatus (open field size 44 × 44 cm) with corresponding software (TSE ActiMot) were used. The dependent variable was distance moved by the animal. Due to apparatus error, one animal from the saline group and one animal from the TMT group were excluded from the analysis.

### 2.6. Histology

On Day 28, the rats were anaesthetized with intraperitoneal injection of ketamine (Narketan, Vétoquinol, Lure, France; 120 mg/kg) and xylazine (Rometar, Bioveta, Ivanovice na Hane, Czech Republic; 6 mg/kg), and transcardially perfused with 0.01 M phosphate buffered saline (pH 7.4) rinse followed by ice cold 4% paraformaldehyde in 0.15 M Na-phosphate buffer and 15% saturated picric acid (pH 7.4). The brains were dissected, postfixed in the paraformaldehyde solution overnight, cryoprotected in buffered 10% and 30% sucrose solution at 4 °C, frozen on dry ice, and stored at −80 °C. The brains were sectioned (coronal plane, 50 µm) using cryostat Leica CM1850. Two series were used for following analyses.

A randomly selected series of sections (every 6th section) was mounted on gelatin-coated slides, stained with cresyl violet, and coverslipped.

Another series of sections (50 µm apart the cresyl violet stained sections) was immunostained with antibodies against neuronal nuclei (NeuN) using the previously described avidin-biotin method [56]. The protocol involved incubation in the solution containing primary antibody (anti-NeuN, mouse monoclonal, clone A60, #MAB377, Chemicon International, Temecula, CA, USA; dilution 1:1000), 1.5% normal horse serum, sodium azide (0.2 mg/mL), and 0.3% Triton-100 in 0.01 M phosphate buffered saline (PBS, pH 7.6) for 72 h at 8 °C. The incubation with secondary antibody was performed using the solution containing biotinylated horse anti-mouse IgG (BA-2001, Vector Laboratories, Burlingame, CA, USA; dilution 1:200), 1.5% normal horse serum, and 0.3% Triton-100 in 0.01 M PBS (1 h incubation at room temperature). After the staining, the sections were mounted on gelatin-coated slides and coverslipped.

### 2.7. Stereological Estimate of CA2/3 Neuronal Density in a Defined Portion of the Dorsal Hippocampus

**Figure 2:**
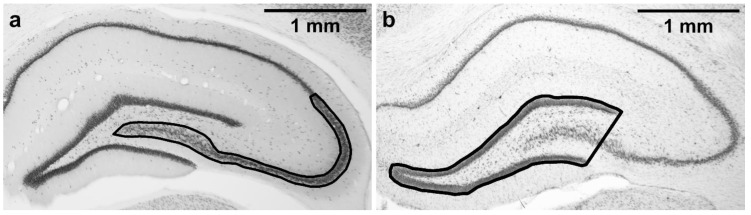
Regions of interest tracing. (**a**) CA2/3 subfield, where the stereological counting was performed; (**b**) area of dentate gyrus. Magnification: 2×. Microphotographs in Figure 2 were acquired using a microscope (Olympus BX53, 2×/0.08 objective lens) connected to a camera (Olympus DP74 for the Olympus BX53 microscope, and Zeiss AxioCam MRm for the Zeiss microscope) and acquisition system in brightfield illumination in grayscale camera mode.

Five NeuN-stained sections starting at the level corresponding to −2.92 from Bregma according to rat brain atlas [57] were used for stereological estimation of neuronal density in the cornu Ammonis 2/3 (CA3 together with CA2) subfield in the dorsal hippocampus. Dorsal CA3 was selected as it is known to be susceptible to TMT-induced damage [20,58] and is also necessary for normal MWM performance [59]. As the CA3/CA2 border is not always easily distinguishable, CA2 was included in the region of interest as well. The region of interest is shown in Figure 2a. Nomenclature (CA3, CA2) is based on Paxinos and Watson [57]. CA3c refers to the portion of CA3 encapsulated by the blades of dentate gyrus, as described in Hunsaker et al. [60].

The neuronal density was determined using unbiased stereology approach. The neurons were visualized with Olympus BX51 microscope (100× oil immersion objective lens) and stereologically counted using optical fractionator [61] using the software Stereo Investigator (MBF Bioscience, Williston, VT, USA). Counting was performed with 50 × 50 μm counting frame and the systematic random sampling grid size was 150 × 150 μm. Tissue thickness was measured at every sampling site, dissector height was 7 μm. Guard zones were used to avoid abnormalities of the tissue surface, guard zone distance was 0.6 μm. Subsequently, estimated population using mean section thickness was calculated by the software. The coefficient of error for a single measurement (Gundersen, m = 1) was ≤0.07. Analysis was performed in a blinded manner.

The volume of the analyzed area (µm^3^) was assessed by the software as well. The neuronal density was then calculated by dividing the estimated neuronal count by the volume of the analyzed area. Right and left hippocampus from each subject was analyzed separately. Subsequently, mean value of neuronal density was calculated for each animal and used for statistical analysis.

### 2.8. Mean Area of Dentate Gyrus in a Defined Portion of the Dorsal Hippocampus

As excessive neurodegeneration in CA3c may lead to thinning of dentate gyrus [62], five cresyl violet-stained sections (corresponding to the NeuN-stained sections used for the stereological counting) were used for dentate gyrus morphometry. Microphotographs were acquired using a microscope (Olympus BX53; 2× objective lens) connected to a camera (Olympus DP74) and acquisition system (Olympus CellSens Dimension 1.18). Analysis was performed using Fiji (ImageJ 2.0.0) software. A line was drawn around the outer borders of the suprapyramidal and infrapyramidal blades of the granule cell layers, and the shape was closed by drawing a straight line connecting the temporal ends of the dentate gyrus blades, resulting in a triangle-like area, including the granule cell layer, a part of CA3 pyramidal layer, and the hilus (Figure 2b). The area of the dentate gyrus was measured. These measurements were done for the left and right hippocampus in all five sections, and the mean of all the 10 measurements per animal was calculated. Analysis was performed in a blinded manner.

### 2.9. Statistics

Statistical analysis was performed using GraphPad Prism 5.0 (San Diego, CA, USA). The differences were considered as significant at *p* < 0.05. Asterisks and number signs in graphs denote statistical significance, * *p* < 0.05, ** *p* < 0.01, *** *p* < 0.001.

Distance moved and mean distance from platform in MWM were analyzed using two-way repeated measures ANOVA (treatment and day factor) followed by Bonferroni post hoc test to assess treatment effect (differences between treatment groups within each day). An additional Bonferroni post hoc test was performed for distance moved to assess the day effect (differences between days within each treatment group).

In the probe trial, the percentage of time spent in the target quadrant was compared to 25% (the value corresponding to random preference for quadrants) using one-sample *t*-test.

The data from MWM visible platform, MWM cumulative latency, dorsal CA2/3 neuronal density, and area of dentate gyrus were tested for normality using Kolmogorov–Smirnov test and for equal variances by Bartlett’s test. If these assumptions for ANOVA were met, ANOVA followed by Tukey’s post hoc test (when appropriate) was used for data analysis (CA2/3 neuronal density, area of dentate gyrus; data are graphically represented as group mean + SEM). If the assumptions for ANOVA were not met (MWM visible platform, MWM cumulative latency), the data were analyzed using Kruskal–Wallis test followed by Dunn’s multiple comparison test where appropriate, and graphically represented as median with interquartile range. The data from fear conditioning (cumulative duration of freezing) were analyzed using ANOVA. The distance moved in open field was analyzed using ANOVA (individual days separately) and, if appropriate, Bonferroni post hoc test was performed to compare the groups with the saline group.

## 3. Results

### 3.1. Morris Water Maze

**Figure 3:**
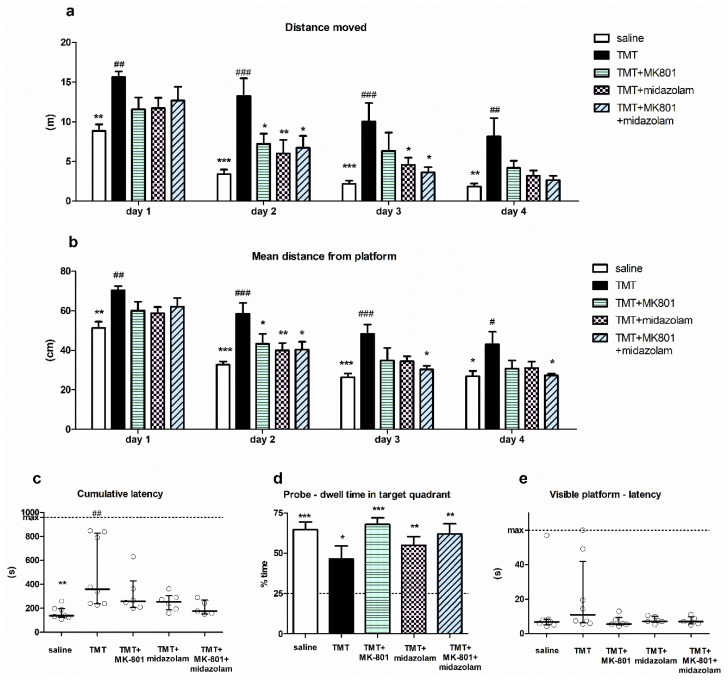
Morris water maze. (**a**) Distance moved. Group mean + SEM, two-way repeated measures ANOVA and Bonferroni post hoc test (treatment effect within day), * *p* < 0.05, ** *p* < 0.01, *** *p* < 0.001 vs. TMT, ^##^ *p* < 0.01, ^###^ *p* < 0.001 vs. saline. (**b**) Mean distance from platform. Group mean + SEM, two-way repeated measures ANOVA and Bonferroni post hoc test (treatment effect within day), * *p* < 0.05, ** *p* < 0.01, *** *p* < 0.001 vs. TMT, ^#^ *p* < 0.05, ^##^ *p* < 0.01, ^###^ *p* < 0.001 vs. saline. (**c**) Cumulative latency. Kruskal–Wallis with Dunn’s multiple comparison test, ** *p* < 0.01 vs. TMT, ^##^ *p* < 0.01 vs. saline. Median with interquartile range, circles represent values from individual animals. (**d**) Probe trial—dwell time in target quadrant. Group mean + SEM, asterisks denote difference from the quadrant choice equivalent to random chance (25%, indicated by the dashed line) analyzed using one-sample *t*-test, * *p* < 0.05, ** *p* < 0.01, *** *p* < 0.001. (**e**) Visible platform trial—latency to find the platform. Median with interquartile range, circles represent values from individual animals.

Spatial cognition of the animals was tested using MWM. Two-way repeated measures ANOVA of distance moved in MWM revealed a significant effect of treatment (F_4,81_ = 6.234, *p* = 0.0011) and day (F_3,81_ = 60.22, *p* < 0.0001), with no interaction. Bonferroni post hoc test (treatment effect) showed that the distance moved by the TMT group was significantly longer than that of saline-treated animals on all MWM days (*p* < 0.01, *p* < 0.001, *p* < 0.001, and *p* < 0.01 for MWM Day 1, 2, 3 and 4, respectively), indicating impaired cognitive performance. Conversely, the distance moved by the treated groups (TMT + MK-801, TMT + midazolam, TMT + MK-801 + midazolam) did not differ from saline-treated animals throughout whole experiment. Moreover, the distance moved by animals treated with TMT + MK-801 on MWM Day 2 was significantly shorter than that of the TMT group (*p* < 0.05). Similarly, on Day 2 and 3, the animals from the TMT + midazolam group (*p* < 0.01 for day 2 and *p* < 0.05 for day 3) and animals from the TMT + MK-801 + midazolam group (*p* < 0.05 for both days) travelled shorter distances compared to the TMT group (Figure 3a). These findings suggest alleviation of TMT-induced cognitive deficit.

Analysis of day effect using Bonferroni post hoc test revealed that the distance moved by saline-treated animals as well as by all groups treated with tested drugs (TMT + MK-801, TMT + midazolam, TMT + MK-801 + midazolam) significantly decreased on Day 2 (compared to the same group on day 1; *p* < 0.001, *p* < 0.05, *p* < 0.01, and *p* < 0.01, respectively), indicating successful learning. In contrast, in the TMT group, there was no significant difference between distance moved on Day 1 and 2. The improvement occurred on Day 3 (vs. day 1; *p* < 0.001), indicating a delayed onset of learning. These findings may further corroborate a beneficial effect of both drugs and their combination on cognition.

Two-way repeated measures ANOVA of mean distance from the platform yielded a significant effect of treatment (F_4,81_ = 5.973, *p* = 0.0014) and day (F_3,81_ = 119.1, *p* < 0.0001). TMT treatment was associated with an increased mean distance from the platform compared to saline on all MWM days (Bonferroni post hoc test, treatment effect, *p* < 0.01, *p* < 0.001, *p* < 0.001, and *p* < 0.05 for Day 1, 2, 3, and 4, respectively). The groups treated with tested drugs did not differ from saline; moreover, they displayed lower mean distance from the platform than TMT-treated rats on certain days: the TMT + MK-801group differed from TMT on Day 2 (*p* < 0.05), TMT + midazolam on Day 2 (*p* < 0.01), and TMT + MK-801 + midazolam differed from TMT on Days 2, 3, and 4 (*p* < 0.05 for all; Figure 3b).

Cumulative latencies (sum of latencies from MWM day 3–4) of the treatment groups were significantly different (Kruskal–Wallis test, H = 14.25, N_1_ = 7, N_2_ = 8, N_3_ = 6, N_4_ = 6, N_5_ = 5, *p* = 0.0065). TMT-treated animals had higher cumulative latency than saline-treated animals (Dunn’s multiple comparison test, *p* < 0.01), indicating impaired performance. Although we observed differences between the group means, with that of saline group being the lowest, followed by TMT + MK-801 + midazolam group, no other statistically significant differences were found (Figure 3c). In the TMT group, three out of eight animals (37.5%) displayed considerably high cumulative latency. High cumulative latency was associated with an increased time spent in the periphery of the pool in these three animals (time spent in the periphery on Day 3–4 was 66–78%, representing the highest values of all animals), suggesting that they failed to adopt effective strategy. One of the six animals (16.7%) in the TMT + MK-801 group tended to manifest similar deficit; however, other groups (saline, TMT + midazolam, TMT + MK-801 + midazolam) were free of such “poor performers”.

Analysis of probe trial showed that despite observed differences in the groups’ performance, animals from all treatment groups displayed significant preference for the target quadrant, as one sample *t*-test revealed that time spent in target quadrant was >25%, which corresponds to random choice (t_6_ = 7.929, *p* = 0.0002 for saline, t_7_ = 2.668, *p* = 0.0321 for TMT, t_5_ = 10.61, *p* = 0.0001 for TMT + MK-801, t_5_ = 5.544, *p* = 0.0026 for TMT + midazolam, t_4_ = 5.728, *p* = 0.0046 for TMT + MK-801 + midazolam group). Therefore, all groups were able to eventually learn the location of target quadrant (Figure 3d).

Finally, we did not detect any significant differences between groups in the visible platform trial. This suggests a low risk of confounding the MWM results via alterations in sensorimotor functions or motivation (Kruskal–Wallis test; Figure 3e).

### 3.2. Contextual Fear Conditioning

**Figure 4:**
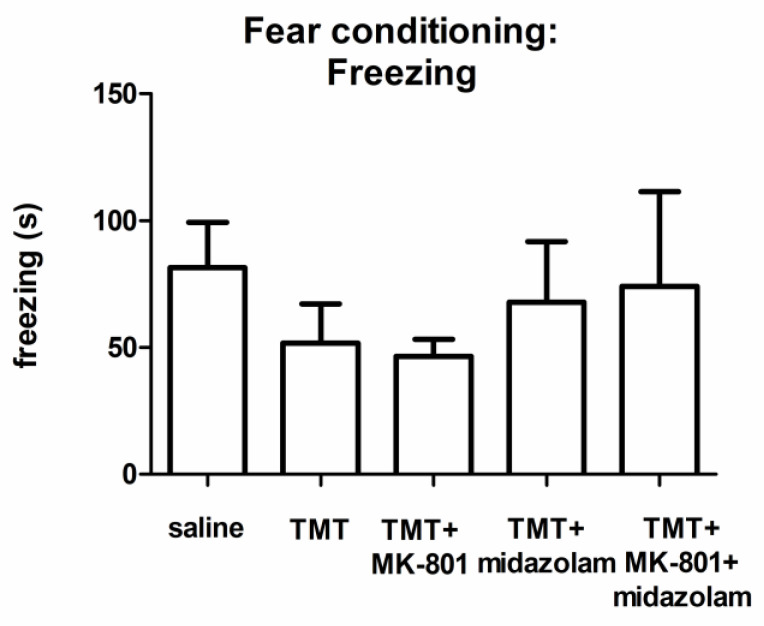
Contextual fear conditioning: Cumulative duration of freezing. Group mean + SEM. No statistically significant differences were found (ANOVA).

According to visual observation during the learning phase, control as well as TMT-treated animals exhibited the typical response to the painful stimulus (running, vocalization; not quantified). ANOVA of the cumulative duration of freezing during the testing phase did not find a statistically significant group effect, although we observed differences in the groups’ means (Figure 4).

### 3.3. Open Field Tests

Analyses of distance moved in open field (Day 0, Day 14, Day 21) did not reveal significant differences between groups (ANOVA). On Day 7, there was a group effect (F_4,25_ = 3.426, *p* = 0.0230), but the post hoc test (Bonferroni) did not find statistically significant differences. Therefore, we failed to detect any significant activity decrease, which would suggest severe impairment of health condition by the toxic effects of TMT (data not shown).

### 3.4. Stereological Estimate of CA2/3 Neuronal Density in a Defined Portion of the Dorsal Hippocampus

**Figure 5:**
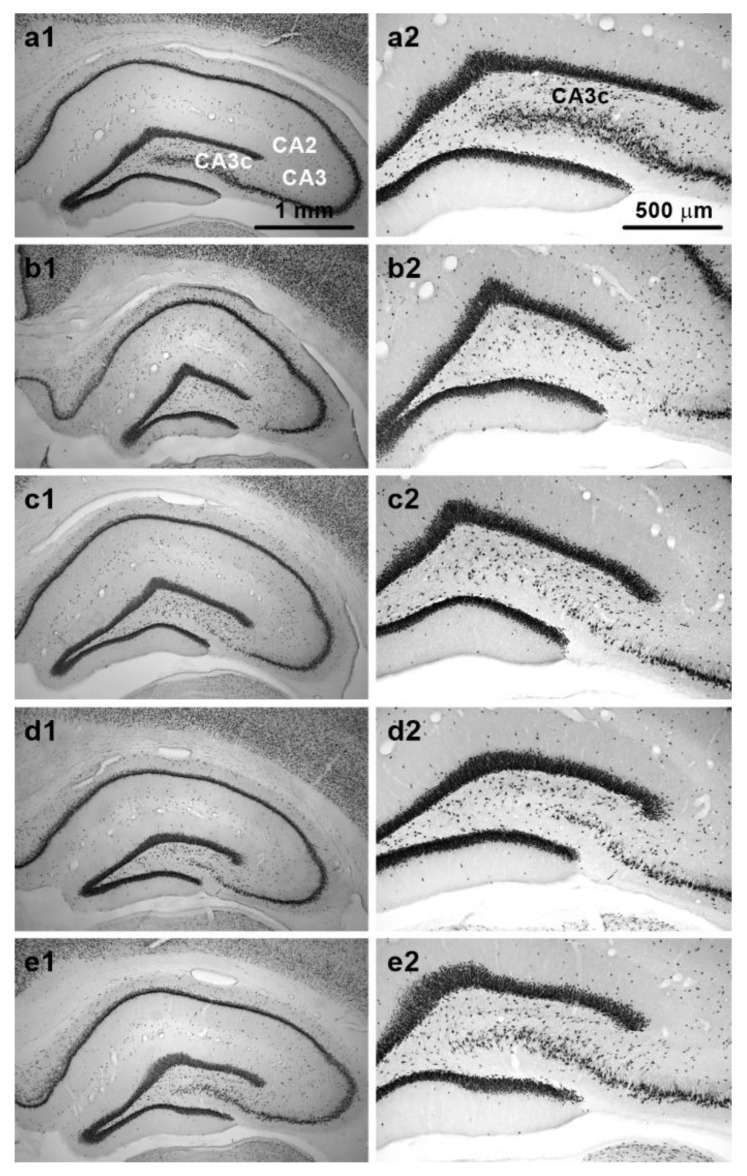
Representative NeuN-stained sections from dorsal hippocampus. Panels show sections from animals treated with: (**a1**,**a2**) Saline (relevant subfields of pyramidal cell layer are marked); (**b1**,**b2**) TMT (note almost complete neuronal loss in CA3c); (**c1**,**c2**) TMT + MK-801; (**d1**,**d2**) TMT + midazolam; (**e1**,**e2**) TMT + MK-801 + midazolam. Note different neuronal densities in CA3c. Sections from animals with neuronal density close to the group mean are depicted. Magnification: 4× (left panels); 10× (right panels). Microphotographs in Figure 5 were acquired using a microscope (left panels: Zeiss Axio Observer D1, 4×/0.1 objective lens; right panels: Olympus BX53, 10×/0.40 dry objective lens) connected to a camera (Olympus DP74 for the Olympus BX53 microscope, and Zeiss AxioCam MRm for the Zeiss microscope) and acquisition system in brightfield illumination in grayscale camera mode.

Examination of NeuN-stained sections confirmed that TMT induced pyramidal cell loss in dorsal CA3, which was particularly prominent in the CA3c subfield. Various degrees of cell loss were observed in individual animals. Representative images of NeuN-stained sections from animals in different treatment groups are shown in Figure 5a1–e2.

**Figure 6:**
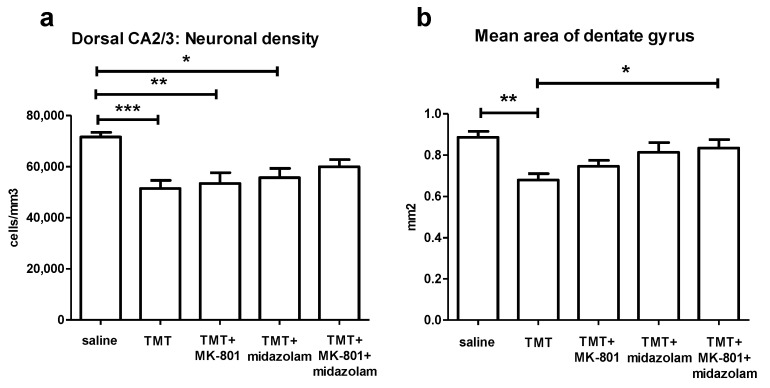
Histology. (**a**) Stereological estimate of neuronal density in CA2/3 in the defined portion of the dorsal hippocampus. Group mean + SEM. ANOVA and Tukey’s post hoc test, * *p* < 0.05, ** *p* < 0.01, *** *p* < 0.001. (**b**) Mean area of dentate gyrus (including the hilus, as described in Methods) in the defined portion of the dorsal hippocampus. ANOVA and Tukey’s post hoc test, * *p* < 0.05, ** *p* < 0.01.

ANOVA of the stereological estimates of neuronal density in CA2/3 in the defined portion of the dorsal hippocampus found significant effect of treatment (F_4,27_ = 6.772, *p* = 0.0007). The TMT (*p* < 0.001), TMT + MK-801 (*p* < 0.01), and TMT + midazolam (*p* < 0.05) group displayed significantly lower neuronal densities than the saline-treated group (Tukey’s post hoc test). In contrast, the TMT + MK-801 + midazolam group did not differ from the saline or from the TMT group, suggesting mild neuroprotective effect (Figure 6a).

### 3.5. Mean Area of Dentate Gyrus in a Defined Portion of the Dorsal Hippocampus

To assess thinning of dentate gyrus, possibly associated with degeneration of CA3c neurons, area of dentate gyrus was measured. ANOVA of the mean area of dentate gyrus revealed significant effect of treatment (F_4,27_ = 5.978, *p* = 0.0014). TMT reduced the area of dentate gyrus (*p* < 0.01, compared to saline, Tukey’s post hoc test). In contrast, TMT + MK-801 + midazolam treatment was associated with increased area of dentate gyrus compared to TMT group (*p* < 0.05), being suggestive of protective effect. The TMT + MK-801 and TMT + midazolam groups did not differ from saline or from TMT group, which may be interpreted as possible partial protective effect (Figure 6b).

## 4. Discussion

The research of the treatment options addressing the disabling cognitive deficit accompanying neurodegenerative diseases is of particular importance. One possible approach may be based on restoration of balance in neurotransmitter systems. The administration of TMT to laboratory rats is considered as a promising model of neurodegenerative diseases associated with cognitive deficit, especially Alzheimer’s disease [35]. The TMT-induced pathologies may involve increased levels of glutamate [30,31] and decreased levels of GABA [23]. Correspondingly, THIP (gaboxadol), representing an agonist of extrasynaptic GABA_A_ receptors [38,63], and phencyclidine, acting predominantly but not exclusively as an NMDA receptor antagonist [37], were found to ameliorate the TMT-induced cognitive deficit. Besides, the co-application of NMDA receptor antagonists with positive modulators of GABA_A_ receptors is associated with beneficial synergistic interactions in other models [15,43,44,45]. This prompted us to investigate the possible benefit of the co-application of these agents in the TMT-induced model of cognitive deficit in rats. MK-801 was chosen as a selective NMDA receptor antagonist [46]. We selected midazolam as a representative of water-soluble [47] GABA_A_ receptor potentiators of the benzodiazepine class.

In accordance with existing literature [23,26], TMT induced cognitive deficit in MWM, manifested as increased distance moved and mean distance from platform throughout the experiment. Moreover, the onset of learning was delayed and TMT-treated animals never reached the level of performance of control animals, as indicated by cumulative latency [54]. According to visible platform trial and open field test, the impaired performance was not caused by non-cognitive phenomena (sensorimotor dysfunction or decreased activity/malaise, respectively). Treatment with MK-801, midazolam, as well as a combination thereof, provided a similar degree of alleviation of manifestations of cognitive impairment in the observed parameters. Our results confirm the protective effects of NMDA receptor inhibition [37] and GABA_A_ receptor positive modulation [38] against TMT-induced cognitive deficit. However, we failed to bring forward any clear evidence for the superiority of the combined treatment. Interestingly, the combined treatment (TMT + MK-801 + midazolam), but not MK-801 or midazolam alone (TMT + MK-801, TMT + midazolam), improved the search strategy on MWM Day3–4, as indicated by the mean distance from the platform [53]. We also observed mildly improved cumulative latency in animals with the combined (TMT + MK-801 + midazolam) treatment compared to the compounds alone; nonetheless, the difference did not attain statistical significance. It is therefore possible that a subtle difference in cognition was present, but that the basic version of MWM used in our study was not sensitive enough to detect it. Anyway, no statistically significant advantage of the combined treatment over monotherapy was found.

As opposed to MWM, we did not find any statistically significant difference between the performance of the treatment groups in the contextual fear conditioning, although we observed tendency to impaired performance in the groups TMT and TMT + MK-801. Hence, our results do not corroborate the findings of Takahashi, who reported decreased freezing in TMT-treated rats in the testing phase of contextual fear conditioning [64]. However, it is worth noting that the experimental design was not identical.

In addition to cognitive aspects, we assessed the TMT-induced hippocampal neuronal loss. Brain samples were harvested with time delay, after completing the behavioral tests (therefore 17 days after treatment cessation). Neuronal loss in CA3 subfield in dorsal hippocampus, a region highly susceptible to TMT-induced damage [20,58], was evaluated using the unbiased stereology approach. Consistently, TMT induced neuronal loss in dorsal CA2/3. Moreover, as the CA3c subfield in the dorsal hippocampus is extremely vulnerable to TMT-induced neurodegeneration [19,20], possibly leading to dentate gyrus thinning [62], the area of dentate gyrus was measured as well, revealing a shrinkage of dentate gyrus in TMT-treated animals. Combined treatment (TMT + MK-801 + midazolam) was associated with mild mitigation of dorsal CA2/3 neuronal loss as well as of dentate gyrus shrinkage, while the substances were largely ineffective when administered alone (TMT + MK-801, TMT + midazolam), suggesting the possible benefit of the combined treatment. It should be noted that the protective effect of the combined treatment at the selected time point was minor. Nevertheless, the analyzed area included the CA3c, the subfield considered the most sensitive for the toxic effects of TMT [19,20], and the beneficial effect of treatment was present even after the 17-day drug-free period. Therefore, although MK-801 [39] or GABA_A_ receptor potentiator phenobarbital [41] alone were reported to fail to protect neurons against TMT-induced degeneration, our results seem to suggest that co-application of similar agents may provide some degree of protective effect.

To sum up, we hypothesized that NMDA receptor antagonists and GABA_A_ receptor potentiators might by complementary mechanisms restore the essential balance between excitation and inhibition in the central nervous system, resulting in desired increase of anti-amnestic effect. However, our results do not support this hypothesis. The behavioral and delayed histological assessments brought different results, with only the latter suggesting possible benefit of combined treatment. It should be emphasized that the benefit of the combined treatment on the histological parameters at selected time point was only minor, and, on the other hand, a non-significant tendency towards improved performance of the group with combined treatment (compared to monotherapy) in Morris water maze was observed.

An insight into the mechanisms underlying the effects of MK-801, midazolam, and their combination in the TMT model is difficult to gain due to the complex and not fully understood mechanisms of action of TMT. In this context, it is challenging to interpret the lack of beneficial interactions of MK-801 and midazolam with respect to the cognitive performance. Moreover, the anti-amnestic and neuroprotective effects of agents interfering with neurotransmitter systems in the TMT model may not be necessarily exerted via identical mechanisms and their relationship is unclear. In general, similar drugs seem to be effective in terms of mitigation of cognitive deficit, but less effective in preventing TMT-induced neuronal loss [36,37,38,39,41]. Addressing the question whether the nature of the anti-amnestic effect is mainly symptomatic or causal is not the objective of the current study and it is also constrained by the design of the experiment, in which, due to ethical reasons, the same animals were used for behavioral and histological assessment (resulting in time gap between them).

Among putative mechanisms underlying the observed mild neuroprotective effect, mitigation of glutamate excitotoxicity seems possible. Excitotoxicity refers to overactivation of glutamate receptors including NMDA receptors by glutamate, enabling calcium influx into neurons, triggering multiple processes including oxidative stress, ultimately resulting in neuronal loss [9]. NMDA receptor antagonists [9,65] and GABA_A_ receptor potentiators [66,67] can mitigate glutamate excitotoxicity and exert neuroprotective effects in other models, and co-application of these agents may increase these effects [15]. Although TMT does not directly activate glutamate receptors [68], glutamate excitotoxicity has been indeed suggested to participate in its neurotoxic effect. As proposed in the 1980s, degeneration of CA3 pyramidal neurons was ascribed to their putative hyperstimulation caused by disinhibited dentate granule cells [20,69,70]. Proven increased release and reduced uptake of glutamate in the TMT model [30,31] may be consistent with this hypothesis. Nevertheless, the role of glutamate excitotoxicity in the TMT model remains controversial [36,39,40] and if it is involved at all, it most likely represents only one of the factors contributing to the neuronal injury, rather than the exclusive one [35,40]. As opposed to excitotoxicity, involvement of oxidative stress in the mechanism of action of TMT is much more definite [35,36]. Activation of NMDA receptors by glutamate may perhaps only potentiate the oxidative effect induced by distinct mechanisms and accelerate the neuronal degeneration [40]. In this context, the antioxidant effect of midazolam [17] seems especially relevant.

In summary, co-application of MK-801 and midazolam did not significantly improve the pro-cognitive effect. Since the mitigation of behavioral and cognitive symptoms in patients is more important than histological aspects, we consider behavioral outcomes of primary importance. From this perspective, MK-801 and midazolam co-application did not lead to an improved effect in the current experimental setup.

## 5. Conclusions

In conclusion, MK-801, midazolam, as well as the combination thereof, mitigated cognitive deficit in MWM; however, we failed to detect any significant superiority of the combined treatment. According to the delayed histological assessment of the neuronal loss in the dorsal CA2/3 hippocampal subfield, a minor protective effect of the combined treatment with MK-801 and midazolam was present, while no significant effect of MK-801 or midazolam alone was detected at the selected time point.

## Data Availability

The data presented in this study are openly available in Mendeley Data at http://dx.doi.org/10.17632/wn442rdyb3.1 (accessed on 19 March 2021).
